# A survey of straw use and tail biting in Swedish pig farms rearing undocked pigs

**DOI:** 10.1186/s13028-016-0266-8

**Published:** 2016-12-05

**Authors:** Torun Wallgren, Rebecka Westin, Stefan Gunnarsson

**Affiliations:** Department of Animal Environment and Health, Swedish University of Agricultural Sciences, PO-Box 234, 532 23 Skara, Sweden

**Keywords:** Intact tail, Manure handling system, Farmer survey, Nursery herd, Finishing herd, Growing pigs, Fattening pigs, Tail docking, Enrichment, Manipulable material

## Abstract

**Background:**

Tail biting is a common problem in intensive pig farming, affecting both welfare and production. Although routine tail docking is banned within the EU, it remains a common practice to prevent tail biting. Straw as environmental enrichment has been proposed as an alternative to tail docking, but its effectiveness against tail biting and function in manure handling systems have to be considered. The aim of the study was to survey how pigs with intact tails are raised and how tail biting is handled in Sweden, where tail docking is banned through national legislation. The study emphasises straw usage and its association with tail biting pigs and problems in the manure handling system. The expectation is that this information could be conveyed to the rest of the EU to reduce the need for tail docking.

**Results:**

In a telephone survey of randomly selected Swedish pig farmers (46 nursery and 43 finishing pig units) with at least 50 sows or 300 finishing places, it was found that straw was used by 98% of the farmers. The median daily straw ration provided was 29 g/pig for nursery and 50 g/pig for finishing pigs in systems with partly slatted flooring. The reported prevalence of tail biting was 1.6% at slaughter. The majority of farmers reported that they never had manure handling problems caused by straw (56% of nursery units and 81% of finishing pig units). A proportion of farmers (37%) also provided with additional material apart from straw on some occasions, which may have affected tail biting prevalence and manure handling problems.

**Conclusions:**

Swedish farmers rear undocked pigs without large problems with tail biting. Straw is the main manipulable material used, and additional manipulable material is used to various extents. The low incidence of straw obstructing the manure handling systems implies that it is indeed possible to use straw in partly slatted flooring systems, reducing the need for tail docking. The impact of using additional manipulable material is unknown and requires more investigation to separate the impact of such material from the impact of straw.

**Electronic supplementary material:**

The online version of this article (doi:10.1186/s13028-016-0266-8) contains supplementary material, which is available to authorized users.

## Background

Tail biting is a common problem in pig production, affecting animal health and welfare, as well as production and profitability [1–4]. In housing systems where pigs have no or limited access to manipulable material, exploratory behaviour can be redirected towards tails, and tail biting may occur [2, 5]. This behaviour can lead to the development of lesions and cause infections so severe that subjected pigs must be prior to slaughter [6, 7]. Reduced welfare caused by tail biting is hence not only attributed to the bitten pig but also to the biting pig, as the behaviour may arise when behavioural needs are unfulfilled [8].

The cause of tail biting is multifactorial, and outbreaks often affect several pigs in the same batch [4, 9]. Identification of specific factors triggering tail biting can be difficult, as triggering factors may differ between outbreaks both within and between farms [4]. However, outbreaks often seem to be associated with housing or management changes such as a new shipment of feed, inadequate indoor climate, season, disease outbreaks or lack of rooting material [1, 3, 9, 10].

Previous studies have shown that providing manipulable material such as straw to growing pigs reduces the incidence of tail biting [2]. Few studies have investigated the amount of straw needed to reduce tail biting. One study showed that pigs provided with more than 100 g straw/pig per day spent less than 5% of their time in redirected behaviours (nibbling another pig; ear- or tail-biting) [11]. Another study suggested that a straw ration exceeding 400 g/pig per day was required to meet the pigs’ need to explore and to reduce oral manipulation of pen mates to a minimum [2]. It is possible that there is a difference between a straw ration that fulfils the pigs’ behavioural need and a straw ration that keeps oral manipulation, such as tail biting behaviour, at an acceptable level.

The EU Council Directive 2008/120/EC, which establishes minimum standards for the protection of pigs, states that “pigs must have permanent access to a sufficient quantity of material to enable proper investigation and manipulation activities, such as straw, hay, wood, sawdust, mushroom compost, peat or a mixture of such […]”. However, the EU directive does not offer any guidance as to how the manipulable materials should be provided to the pigs (i.e., how much or how frequently) and a large variation can therefore be expected. Furthermore, knowledge of how much straw is needed to fulfil the legislative requirements concerning proper investigation or manipulation activities is limited.

Routine tail docking is banned within the EU according to The EU Council Directive 2008/120/EC. Still, over 90% of pigs in the EU are tail docked, especially pigs raised on slatted flooring [12]. Sweden, as well as Finland and Lithuania, is an exception; 100% of the pigs are reared with intact tails due to a total ban on tail docking by national legislation [12, 13].

In addition, the space allowance in Swedish pig farms is higher compared to the minimum requirements of the EU directive. For example, the space requirements per nursery pig of 20 kg live weight (LW) are 0.32 m^2^ in Sweden compared to 0.20 m^2^ according to the EU legislation. For a finishing pig of 110 kg LW, the space requirement is 1.02 m^2^ in Sweden and 0.65 m^2^ according to the EU legislation [11, 13]. The maximum amount of slatted floor is weight dependent in Sweden. Maximum amount of slatted flooring range from 32 to 25% for pigs between 20 and 110 kg, e.g. 32% for 20 kg LW pigs and 25% for 110 kg LW, while there is no ban on fully slatted floors for growing pigs in the EU. The maximum widths of slat openings are 14 mm for nursery pigs (9–25 kg LW) and 18 mm for finishing pigs (>25 kg LW); the minimum slat widths are 50 mm for nursery and 80 mm for finishing pigs according to both Swedish and EU legislation [13, 14].

Swedish pig farmers may possess valuable information regarding the rearing of undocked pigs and how to use straw in partly slatted flooring systems. As far as we know, this information has not yet been systematically documented in Sweden. The expectation is that this information could be conveyed to the rest of the EU to reduce the need for tail docking.

The aim of this study was to describe the main management factors in rearing undocked pigs in Sweden and how tail biting is handled. The study emphasises straw usage and how straw is associated with bitten pigs and problems in the manure handling systems.

## Methods

### Farmers and data records

A telephone survey of commercial pig farmers in Sweden was conducted from July to November 2014. Their contact information was supplied in January 2014 by the Swedish Board of Agriculture. To be included on the contact list, farms had to have at minimum 50 sows or 300 finishing pig places. The farms were categorised as nursery farms (keeping nursery pigs approximately 5–12 weeks of age; 10–30 kg LW), finishing pig farms (keeping growing pigs from approximately 12 weeks of age until slaughter around 6 months of age; 30–120 kg LW) or integrated farms keeping both nursery and finishing pigs. The final contact list consisted of 747 nursery farms, 892 finishing pig farms and 4618 integrated farms. Farmers were called in a random order generated by a computer.

All telephone calls were conducted by a single person according to the randomised list. The farmers were informed of the purpose of the survey and told it was being conducted on behalf of the Swedish University of Agricultural Sciences. Participation was voluntary, and reasons for not participating were recorded. Each farmer not answering the phone was called a maximum of three times before being excluded as “unable to reach”.

### The survey

The survey consisted of two general questions, 60 questions (35 multiple-choice, 25 open-ended) related to nursery pigs and 28 questions (29 multiple-choice, 29 open-ended) related to finishing pigs. Farmers with integrated farms answered questions concerning nursery and finishing pigs separately. The complete survey is presented in the Additional file 1, and the layout of the survey was as follows:

- General questions—e.g. type of production (conventional or organic) and breed.
- Production information—number of pig places and growth rate.
- Tail biting—e.g. occurrence of bites (frequency, amount), suspected causes.
- Straw usage—e.g. species, ration, frequency of straw and/or other manipulable material provision.
- Pen conformation—e.g. pen size, type of flooring.
- Feeding system—e.g. type of feed, feeding system.
- Manure handling—e.g. type of manure handling system, occurrence of problems related to straw usage.

### Calculation of straw usage

A majority of farmers (72%) reported straw usage as the number of bales used in a week or in a year combined with bale weight. The amount of straw provided per pig/day in kg could hence be calculated according to the following equations:

$$\begin{aligned} &\frac{{\left( {\frac{{{\text{straw usage per week}} ({\text{kg}})}}{\text{number of nursery piglet places in the herd}}} \right)}}{7} \\ &\quad = {\text{straw ration}}/{\text{nursery pig per day (kg)}} \end{aligned}$$

$$\begin{aligned} &\frac{{\left( {\frac{{{\text{straw usage per week }}({\text{kg}})* 52\;{\text{weeks}}}}{\text{number of produced finishing pigs per year}}} \right)}}{{99^{a}}} \\ &\quad = {\text{straw ration}}/{\text{finishing pig per day (kg)}}\end{aligned}$$

According to the Swedish national herd monitoring database (PigWin Slakt), the average number of days (^a^) in the finishing pig unit for rearing a finishing pig was 99 days in Sweden in 2014 [15].

Integrated farms were divided into nursery and finishing units. The survey contained no information regarding the number of batches or pens per unit. Thus, to acquire a relevant estimation of straw use, the calculations of straw use were based on the total number of nursery pig places or finishing pigs produced per year.

### Statistical analysis

The information obtained in the survey was recorded in Microsoft Excel and transferred to SAS software ver. 9.4 (SAS Inst. Inc., Cary, NC, USA) for statistical analysis. Descriptive statistics were calculated and presented as frequencies, and mean and median values. Because data were not normally distributed, median values were preferred over mean values.

Outcomes from the questions in the survey were classified as either continuous (e.g., straw usage), ordinal (possible to rank; e.g., frequency of tail biting estimated by the farmer) or categorical (not possible to rank, e.g., strategies for avoiding outbreaks of tail biting). Categorical data were solely presented as frequencies and median/mean when feasible.

Correlations between continuous and ordinal outcomes were analysed with Spearman’s rank correlation, as the data were not normally distributed. Correlations were calculated for variables where there was information about both parts of the investigated correlations, namely:

- Straw ration and frequency of problems in the manure handling systems (including and excluding deep litter systems).
- Frequency of tail biting and affected pens/outbreak, percentage of bitten pigs/batch at slaughter, number of slaughtered pigs per year and straw ration.

## Results

### Response rate and general information about participating farms

A total of 139 farmers were called. Out of these farmers, 60 participated in the survey (43% response rate); 28 (20%) could not be reached; six (4%) did not have the time to participate; six (4%) were unwilling to participate and 38 (28%) did not have pigs anymore or did not have pigs according to the inclusion criteria of the study. The participating farmers included 29 integrated farms, indicating that in total, 46 farmers answered the nursery pig part of the survey and 43 farmers answered the finishing pig questions. Seven of the participating farms produced organic pigs. One survey was excluded because it was incomplete due to technical problems.

Detailed information about participating farms is found in Table 1. The breeds used were Landrace (L)*Yorkshire(Y)*Hampshire crosses (35), L*Y*Duroc-crosses (24), L sows (3), Y sows (3), LY sows (2), and Linderödsvin (Swedish native breed) (1). Eight farmers kept several breeds within the same farm.

**Table 1:** General information of participating pig farms, reported by farmers

Parameter	Nursery units						Finishing pig units					
	n	Median	Mean	SD	Min	Max	n	Median	Mean	SD	Min	Max
Total number of pig places	46	815	1088	894	126	4000	43	1200	1434	1164	132	6500
Number of produced pigs/year	–	–	–	–	–	–	43	3600	4484	3795	350	20,000
Age at entry (days)	35	35	37.9	8.9	31	84	42	84	84	8.7	63	105
Weight at entry (kg)	35	10.5	11.4	5.1	9	40	43	30	31	2.8	25	37
Number of pigs/pen	45	11	24	43	10	300	43	10	15	17	7	100
Size of pen (m^2^)	45	6	21	49	3	300	43	9.6	19	40	7	250
Space allowance/pig (m^2^)	44	0.5	0.53	0.13	0.3	1.9	43	1	0.96	0.08	0.8	2.5

n is number of farmers answering the question. Eleven of the farmers did not move nursery pigs into a new stable after weaning, which is why age and weight at entry to the nursery unit was only answered by 35 farmers

### Occurrence of bitten pigs and suspected causes

Bitten pigs were reported in 50% of the nurseries and 88% of the finishing pig units. In affected nursery units (n = 23), 78.3% of farmers observed bitten pigs less than two times/year, 17.4% 3–6 times/year and 4.3% at least every month (tail-biting frequency). In affected finishing pig units (n = 38), 21% observed bitten pigs less than two times/year, 37% 3–6 times/year, 34% at least every month and 8% every week (tail-biting frequency).

Farmers reported observing bitten pigs in on average 1.6 pens (median 1, range 1–5) during the last tail biting outbreak in nursery units and in on average 1.5 pens (median 1, range 1–4) in finishing pig units. The finishing pig units had on average 1.6% bitten pigs in each batch at slaughter (range 0.1–6.5%). The number of affected pens per outbreak was positively correlated to the tail-biting frequency (r = 0.33, *P* = 0.04, n = 38) in finishing pig units. The tail-biting frequency was also positively correlated to the percentage of bitten pigs (r = 0.55, *P* < 0.001, n = 38) and to the number of slaughtered pigs/year of the farm (r = 0.32, *P* = 0.05, n = 38), in finishing pig units.

The most commonly reported causes attributed to tail biting outbreaks by farmers were stocking density in nursery farms and stocking density and salt deficiency in finishing pig units (Table 2).

**Table 2:** Suspected causes for tail biting in Swedish pig farms, reported by farmers

	Nursery units n = 23		Finishing pig units n = 38	
	During the last outbreak	Overall on the farm	During the last outbreak	Overall on the farm
Coincidence	8	5	1	0
Do not know	5	3	11	10
Feed composition/feed equipment	0	0	12	15
Genetics/nervous pigs	4	2	8	7
Insufficient feeding space	0	0	0	5
Insufficient ventilation/high temperature	0	0	4	7
Lack of occupation	1	1	0	0
Salt deficiency	0	7	0	0
Stable/climate	0	1	0	0
Stocking density/overcrowding	6	6	2	5

n is number of farmers answering the question

Coincidence: e.g. a pig escaping into the wrong pen

Only farmers that had observed tail biting on their farm (23 nursery farmers and 38 finishing pig farmers) answered the questions about causes for tail biting outbreaks. Each farmer could give several causes for tail biting outbreaks, which is why the number of causes could exceed the number of respondents

### Prevention of outbreaks and treatment of bitten pigs

All farmers except in one nursery and two finishing pig units (95%) reported that they took measures to prevent outbreaks after observing bitten pigs. The most common preventive action reported to mitigate an ongoing outbreak was to identify and separate the biting pig (Table 3). Bitten pigs were treated with antibiotics in 76% of the nurseries and 92% of the finishing pig units, while one farmer provided analgesia (Table 4). Farmers in two nursery (4.3%) and four (9.3%) finishing pig units reported that they would have wanted to dock tails if it were legal in Sweden.

**Table 3:** Measures for preventing tail biting outbreaks after observed tail wounds in Swedish pig farms, reported by farmers

	Nursery units n = 21	Finishing pig units n = 38
Addition of extra manipulable material or “toys”	5	5
Checking ventilation/feeding system	5	7
Increasing of straw ration	6	11
Nothing	1	2
Other	4	0
Separation of biter	12	20
Separation of bitten pigs	6	14

n is number of farmers answering the question

Only farmers that had observed tail biting on their farm (23 nursery and 38 finishing pig units) could answer the questions about causes for tail biting outbreaks. Each farmer could give several causes for tail biting outbreaks, which is why the number of causes could exceed the number of respondents

**Table 4:** Treatments of tail bitten pigs in Swedish pig farms, reported by farmers

	Nursery units n = 23	Finishing pig units n = 38
Analgesia/pain killers	1	0
Antibiotics, kept in original pen	13	21
Antibiotics, moved to sick pen	3	14
Moved to sick pen without antibiotics if pig look healthy	0	2
Nothing	3	3
Other	6	4

n is number of farmers answering the question

Only farmers that had observed tail biting on their farm (23 nursery farmers and 38 finishing pig units) could answer the questions about causes for tail biting outbreaks. Each farmer could give several causes for tail biting outbreaks, which is why the number of causes could exceed the number of respondents

### Usage of straw and other manipulable materials

All farmers except two (in one nursery and one finishing pig unit), i.e., 99%, stated that they provided pigs with straw (Table 5). These two farmers reported using sawdust or wood shavings instead, as they believed that straw made pens slippery and caused blockages in the manure handling system. Among farmers that used straw, chopped straw was used in 77% (n = 44) of nursery and 74% (n = 42) of finishing pig units. Straw was the only manipulable material used in 60.9% (n = 46) of nursery and 67.4% (n = 43) of finishing pig units, while the remaining farmers provided additional manipulable material. Usage of additional manipulable material was used to keep pens dry (11), when moving piglets into the nursery unit (3), when it was hot outside (2), to create a light environment (2) and when there was not enough straw (1) in nursery units. In finishing pig units, additional material was used when moving pigs into the finishing unit (7), if pens were wet/dirty (4), to create a light environment (1), to provide enrichment to the pigs (1), when there was not enough straw (1) and because additional straw would cause blockages in the manure handling system (1). Each farmer could give several reasons for using additional material, which is why the number of answers exceeds the number of respondents. Hence, straw was commonly the only provided manipulable material; additional manipulable material was mainly used at special occasions.

**Table 5:** Type of manipulable materials used in Swedish pig farms, reported by farmers

	Nursery units n = 46	Finishing pig units n = 38
	n (%)	n (%)
Straw only	28 (60.9)	24 (63.2)
Straw + saw dust	0 (0)	2 (5.3)
Straw + wood shavings	16 (34.8)	9 (23.7)
Straw + meal	0 (0)	1 (2.6)
Straw + peat + wood shavings	1 (2.2)	1 (2.6)
Saw dust only	1 (2.2)	0 (0)
Sawdust + wood shavings	0 (0)	1 (2.6)

n is number of farmers answering the question

The calculated daily straw rations in systems with partly slatted floorings were median 35 g/pig per day for nursery pigs and 50 g/pig per day for finishing pigs (Table 6). Six nursery and four finishing pig units had deep litter systems, which are very straw intensive. When farms with deep litter systems were excluded, the median straw ratio decreased (Table 6).

**Table 6:** Straw ration and straw length of provided straw in Swedish pig farms

	Nursery units						Finishing pig units					
	n	Median	Mean	SD	Min	Max	n	Median	Mean	SD	Min	Max
All farms (including deep straw bedding)												
Straw length, cm	35	5	6.3	2.6	1	10	31	8	8.2	4.1	1	20
Straw ration, g/pig per day	29	45	99	149	8	714	35	53	130	225	9	1156
Excluding farms with deep straw bedding												
Straw length, cm	35	5	6.3	2.6	1	10	28	6.5	7.8	4.1	1	20
Straw ration, g/pig per day	22	29	35	24	8	85	28	50	62	45.5	9	225

n is number of farmers answering the question

The straw ration per pig was calculated from the farms straw consumption. Farms that were unable to provide such information was excluded from the calculations and hence the number of farms were reduced from 43 to 35 nursery units and 43–31 finishing pig units

The straw ration and straw length was also calculated excluding farms with deep litter systems (removing 6 nursery units, 4 finishing pig units) since deep litter systems are uncommon in European pig production

A proportion of farmers (24%) reported that they would like to increase the straw ration unless it was limited by other factors. The most commonly reported limiting factor preventing farmers from increasing straw rations was that it was thought to block the manure handling system (Table 7).

**Table 7:** Reported limitations to increase straw rations in Swedish pig farms, reported by farmers

	Nursery units, n n = 13	Finishing pig units, n n = 8
Would be too costly	1	1
Would be too dirty	3	2
Would block manure handling system	5	6
Would block slats	5	1
Would require too much work	2	0

n is number of farmers answering the question. Only farmers reporting that they would have wanted to increase the straw ration if possible answered this question and gave reasons for why an increase of the straw ration was impossible

Each farmer could give several causes limiting an increased straw ration, which is why the number of causes could exceed the number of respondents

Objects other than manipulable materials were provided in 13% (n = 46) of the nursery and 16% (n = 43) of the finishing pig units. These other objects were balls (2), rope (3), silage/hay (1), newspapers (1), sacks (6), chains (1), wood logs (5) and salt stones (5). However, in 69% of the units, pigs had access to the additional objects only when bitten pigs had been observed to prevent tail biting and fighting between pigs at certain occasions, such as when pig groups were mixed.

The majority of farmers provided new straw daily (76% of nursery and 83% of finishing pig units) (Table 8). Straw was distributed evenly on the pen floor (41% of nursery and 38% of finishing pig units) or in a pile on the pen floor (59% of nursery and 62% of finishing pig units).

**Table 8:** Frequencies of new straw allocation in Swedish pig farms, reported by farmers

	Nursery units, n (%) n = 45	Finishing pig units, n (%) n = 41
Deep straw bedding, never adding new straw	2 (4.4)	0 (0)
Every second week	0 (0)	1 (2.4)
Once/week	2 (4.4)	1 (2.4)
Twice/week	1 (2.2)	0 (0)
Every second day	1 (2.2)	0 (0)
Once/day	34 (75.6)	34 (82.9)
Twice/day	5 (11.1)	5 (12.2)

n is number of farmers answering the question. Only farmers that reported using straw could answer this question

The ration of manipulable material was modified by farmers depending on the health status in the pen in 35% (n = 46) of the nursery and 33% (n = 42) of the finishing pig units. Some nursery units were given more straw if pigs seemed cold or dirty (5), switched to wood shavings when pigs were sick (7) or switched to peat when pigs had diarrhoea (3). One farmer reported providing less straw when pigs were sick. In finishing pig units, straw rations were increased when bitten pigs were observed (9), when pigs had diarrhoea or it was wet in the pen (3) or when the animals were sick (2). One farmer decreased the ration if it was hot outside. Farmers could give several reasons for altering the manipulable material ration, which is why the amount of answers exceeds the number of responding farmers.

### Straw usage and tail biting

In finishing pig units, the reported occurrence of bitten pigs had a negative rank correlation with deep straw systems (r = −0.42, *P* < 0.01, n = 38) compared to systems that had partly slatted flooring, and there was a negative rank correlation between size of straw ration and tail-biting frequency (r = −0.32, *P* < 0.05, n = 37). In nursery units, the coefficient of correlation between reported tail-biting frequency and straw ration was r = −0.328 (*P* < 0.01, n = 38).

### Manure handling systems

The most common manure handling system reported was rope/cable and arm scrapers (Table 9). When asked how frequently straw caused blockages or other problems with the manure handling system, 56% nursery and 81% finishing pig units had never experienced problems in the manure handling system caused by straw. No farmer reported problems in manure handling more often than once a month. There was a negative rank correlation between frequency of manure handling problems and straw ration in nursery units (r = −0.44, *P* = 0.02, n = 28), but not in finishing pig units (r = 0.16, *P* = 0.35, n = 35). When farms with deep straw bedding were excluded, there was no correlation in nursery units (r = −0.33, *P* = 0.12, n = 22), but a tendency was found in finishing pig units (r = 0.31, *P* = 0.09, n = 31).

**Table 9:** Frequencies of problems caused by straw in manure handling systems used, reported by Swedish pig farmers

	Nursery units, n (%) n = 45					Finishing pig units, n (%) n = 43				
	Total	Never	Few times/year	At least monthly	At least weekly	Total	Never	Few times/year	At least monthly	At least weekly
Rope/cable with arm scraper(s)	23 (51.1)	12 (52.3)	8 (34.8)	3 (13)	0 (0)	25 (58.1)	22 (88)	2 (8)	1 (4)	0 (0)
Rope/cable with box scraper	6 (13.3)	2 (33.3)	3 (50)	1 (16.7)	0 (0)	9 (20.9)	6 (66.7)	3 (33.3)	0	0 (0)
Hydraulic scraper	4 (8.9)	3 (75)	1 (25)	0	0 (0)	1 (2.3)	1 (100)	0	0	0 (0)
Shallow pit with pull-plugs	6 (13.3)	3 (50)	2 (33.3)	1 (16.7)	0 (0)	3 (6.9)	1 (33.3)	2 (66.7)	0	0 (0)
Deep straw bedding	6 (13.3)	5 (83.3)	1 (16.7)	0	0 (0)	4 (9.3)	4 (100)	0	0	0 (0)
Slurry	0 (0)	0	0	0	0 (0)	1 (2.3)	1 (100)	0	0	0 (0)

n is number of farmers answering the question

## Discussion

### Response rate and farmer self-reporting

According to the Swedish agricultural statistical compilation 2016 [16], there were 1228 pig businesses in Sweden in 2015, meaning that 5% of Swedish pig farms are included in this study. All farmers were called in a randomised order. The vast majority of reached farmers that fit into the inclusion criteria (84%) wanted to participate in the study, which indicates that we have a representative sample of Swedish farmers.

The results from this study are based on farmers answering questions and reporting about how pigs with intact tails are reared. Self-reporting of routines and how often bitten pigs are seen is not exact. Farmers report their perceptions, but reality may differ for different reasons such as the farmer not being aware of the *true* prevalence of tail biting outbreaks and reporting only detected outbreaks. The aim of this study was to investigate how undocked pigs are reared, how farmers handle bitten pigs, how straw is managed and how its use is linked to tail biting and manure handling problems. Due to the nature of the aims, a survey in which farmers report their experiences gives more information in this stage than a controlled on-farm study would. The fact that farmers reported similarly (e.g., similar causes of tail biting outbreaks, similar frequency of tail biting outbreaks) indicates that the results are reliable. Furthermore, the results of this study are in accordance with those of previous studies (e.g., number of bitten pigs/batch and that tail biting increases with increased age), which also indicates that the results from this study are reliable. However, the results of this study should be considered for what they are: farmers’ opinions and not absolute facts.

### Occurrence of bitten pigs and suspected causes

In the present study, suspected causes of tail biting varied between farms and pig age groups (nursery or finishing pigs), which is in accordance with previous studies concluding that different production systems have different risks for developing tail biting [9, 10]. Previous findings show that pigs with permanent access to straw are mainly exposed to climatic risk factors for tail biting (e.g., temperature and air humidity), whereas pigs with restricted access to manipulable material were mainly exposed to the risk factor “strawless system” [10].

In the present study, farmers suggested stocking density as the main risk factor in nursery units and feed composition/feed equipment as the main risk factor in finishing pig units and did not suggest factors related to the housing climate, which have also previously been considered less important [10]. This finding is partly in accordance with a survey showing that pig farmers in The Netherlands (rearing docked pigs) consider stocking density to be the main risk factor for tail biting, along with stable climate [17]. Stocking density has been considered to be one of the most common risks associated with tail biting in pigs reared in partly slatted systems with straw, as seen in previous studies [12]. Although the maximum stocking density in Sweden is lower than specified in the EU directive, stocking density was considered the most common cause of tail biting outbreaks in nursery pig units in this study and was also highly ranked in finishing pig units. It has been shown that tail biting significantly increases when the feeding space decreases in nursery units [4] and as the pen facilities remain unchanged, the stocking density will increase in the pens, decreasing feeding space per pig. Therefore, increased stocking density is associated with an increase in other pen-related risk factors. Stocking density and not only provision of additional manipulable material may be an important preventive factor for rearing undocked pigs with low incidences of tail biting.

Only one nursery farmer thought that observed tail biting outbreaks were caused by boredom. This result could indicate that farmers are satisfied with the provided straw ration and outcome. Dutch farmers report similar a prevalence of problems with tail biting (approximately 50% have tail biting problems, ranging from 1 to 6% in docked pigs) but consider provision of manipulable material to be less efficient than docking for preventing tail biting [17]. The manipulable materials used in the Dutch non-organic farms were mainly objects such as chains and ropes, while straw, sawdust or wood shavings were used by only 2–3% of participating farms. Similarly, in a survey of Finnish farmers rearing undocked pigs, manipulable material was considered less important than feeding space, animal health and indoor climate [18], but there was no information about what type of manipulable material was provided daily to the pigs in that study.

It is difficult to evaluate and compare the percentage of bitten pigs between countries and studies due to the different scoring criteria used. A review comparing the prevalence of tail biting from different sources found a wide range both within and between countries depending on data source [19]. This variation may partially be attributed to differences in definition, as definitions range from swollen tails to tissue loss [19, 20]. In this survey, the “bitten” was not defined in the protocol or by surveyed farmers. According to finishing pig farmers, the percentage of bitten pigs was on average 1.6% at slaughter, which is in agreement with tail biting recorded by National Food Agency standards in two Swedish abattoirs (1.5–1.9%) [20]. In addition, the finding that tail biting increases with age is in agreement with the findings of previous studies [3, 21]. We therefore argue that the farmers’ definition of bitten was similar to that used by the National Food Agency: “evident biting damage” or “at least half the tail missing” [22]. According to this definition, only severely bitten pigs were recorded; milder tail lesions without blood that are not easy to detect when looking at the pig from outside the pen were excluded from this study. Both abattoir and farmer scoring may therefore be under-reporting tail lesions compared to a scoring scheme that includes a swollen tail or superficial scratches as the lower limit for bitten [20, 23]. However, according to Swedish regulations, pigs with swollen tails or unhealed tail wounds are not allowed to be transported to the abattoir, which is why these types of injuries might not appear in slaughterhouse data even if the criteria were altered. Also, less severe wounds may be of less importance when investigating the current aim (i.e. how pigs with intact tails are reared and managed). Tail docking is conducted to reduce the problems with injured pigs and carcass losses due to more severe tail wounds. Hence, the lack of information about tail lesions that are less severe than those reported at the abattoir is of less importance for investigating whether or not it is possible to rear pigs with intact tails although less severe wounds may also affect the production and welfare of the pigs [e.g. 12].

### Usage of straw and other manipulable materials

All participating farmers reported providing access to manipulable material; of these farmers, 99% provided straw in various amounts. The wide usage of straw in this study indicates that straw usage is considered feasible and functional under commercial conditions in Sweden.

Permanent access to straw has previously been defined as the permanent presence of more than one litre of unsoiled straw in the pen before new straw allocation [2]. Permanent straw access was achieved at 80–290 g straw/pig per day for 30–80 kg LW pigs at a stocking density of 0.7 m^2^ per pig according to a Danish study [2]. According to this definition, many Swedish farmers do not provide permanent access, as the reported median straw ration was 29 g/pig per day for nurseries and 50 g/pig per day in partly slatted flooring systems.

In this study, however, all calculations regarding daily ration were based only upon the amount of straw, even though 37% of the farmers also provided additional material to various extents. Therefore, the actual ration of accessible manipulable material may be underestimated here. A more detailed analysis of the other manipulable material was not possible because the survey did not request further information on this matter. When developing the survey and collecting experiences from researchers and the Swedish trade organisation for pigs, the wide use of additional material was not foreseen. However, it was shown that provision of additional material was common only during certain periods of time, such as when pigs are newly moved into the stable or due to poor pen hygiene, while straw was given on a daily basis. Additional material may therefore have been of less importance in preventing tail biting outbreaks on participating farms.

If the provided additional material is of high value for the pig, it may affect tail biting behaviour. High-value materials that stimulate pig exploratory behaviour are destructible, manipulable, changeable and complex [7]. Furthermore, the most used objects during the first 24 h after presentation are odorous, deformable, rootable, unattached and chewable according to previous studies [24]. The most used objects 5 days after presentation are ingestible, destructible, contained (e.g., straw provided in a box as opposed to directly on the floor as in this study), particulate and rootable [24]. In this study, sawdust and wood shavings were the most commonly provided complements to straw and they may possess the previously identified characteristics. Additionally, the pigs’ usage of sawdust or wood shavings is reduced when they are combined with another material with greater manipulative value [25], and both chopped and long straw are used more than sawdust [24]. The effect of adding sawdust or wood shavings to straw is therefore limited, and the usage of additional material along with the straw could be considered to have a limited effect on tail biting behaviour.

Straw length has, however, been suggested to affect its investigative value for the pigs, as reduced straw length decreases the diversity of behaviours [26]. The majority of farmers in this study used chopped straw, with a mean length of 5 cm in nursery and 6.5 cm in finishing pig units. This straw is considerably longer and therefore possibly of higher value than the chopped straw of 1–4 cm used in the previously mentioned study [26]. The straw on Swedish farms may still be long enough to be of high investigative value to the pigs and seems efficient in preventing tail biting behaviour.

### Straw usage and tail biting

The amount of straw needed to reduce the prevalence of tail biting behaviour by satisfying the need to explore has previously been determined to be approximately 400 g straw/pig per day for 30–80 kg LW pigs and 400 g/pig per day for what the author describes as nursery-finishing pigs [2, 11]. Again, this ration is far greater than most of the straw rations reported in this study (median 45–53 g/pig per day). However, the reported rates of bitten pigs at slaughter (average 1.6% at slaughter) and tail biting were low, and no bitten pigs were ever seen in 50% of the nursery and 12% of the finishing pig units. Furthermore, the number of affected pens during an outbreak was reported to be low in both nursery and finishing pig units (on average 1 pen/outbreak), indicating that few pigs are affected in each outbreak and thus that the environment is overall sufficient. The use of additional material along with straw and its effect on tail biting was not assessed in this study.

### Straw usage and manure handling

Straw provision in pig pens has been suggested to cause blockage of the slatted floor and to cause obstructions in the manure handling system [6, 27]. However, chopped straw reduces the need for manual cleaning of the pen compared to long straw [25]. Previous studies have shown that provision of large amounts of straw in partly slatted farrowing pens does not cause blockage of slats if straw chop length is adjusted to the type and design of the slatted flooring [28]. The results of this study show that the frequencies of reported problems in the manure handling system were low. The majority of farmers (76%) provided chopped straw, which could explain the low frequency of problems caused by straw in manure handling systems.

The main reported restriction on increasing the straw ration was that straw was thought to cause blockages in the manure handling system or the slatted floor, although most farmers reported never having experienced these problems. This finding implies that the argument that straw would be unusable with current manure handling systems is not correct and shows a gap in knowledge where future research is needed. Straw could also be used in fully slatted flooring systems given that the pigs can make use of the straw before it passes through the slats.

It is reasonable to believe that the pipe diameter of the manure handling systems has an impact on the frequency of obstruction. Pipe diameter is not regulated in the EU Directive or in the Swedish national legislation and could therefore not be assessed in this study, but it could be an interesting area of new research.

## Conclusion

This study surveyed straw usage and tail biting on Swedish pig farms. All interviewed farmers reported supplying pigs with manipulable material. The majority of farmers (99%) used straw in various amounts, and 37% also supplied other materials along with the straw (e.g., wood shavings) on certain occasions. Even though the straw rations were restricted (approx. 50 g/pig per day), the percentage of bitten pigs at slaughter (1.6%) and the tail-biting frequency were low (twice yearly in nursery units and 3–6 times/year in finishing pig units). Straw was commonly provided daily, but the farmers reported limited problems in the manure handling systems. We conclude that tail biting can be prevented by straw access in undocked pig populations and that daily straw usage is possible in commercial pig production.

## Additional file

**Additional file 1.** A survey of straw use and tail biting in Swedish pig farms rearing undocked pigs.
