# Supplementary material for: A survey of straw use and tail biting in Swedish pig farms rearing undocked pigs
Source: Acta Vet Scand. 2016 Dec 5;58:84. doi: 10.1186/s13028-016-0266-8 (PMC5139032; doi:10.1186/s13028-016-0266-8)
Supplement: Supplementary file 1 — Additional file 1. A survey of straw use and tail biting in Swedish pig farms rearing undocked pigs. [file 13028_2016_266_MOESM1_ESM.pdf]

A survey of straw use and tail biting in Swedish pig farms rearing undocked pigs

The survey has been translated from Swedish to English for publishing purposes. The original survey and all interviews were performed in Swedish.

A. **Farm ID:**

B. **Farm ID according to the list:**

C. **Interview date:**

D. **Are you raising certified organic pigs?**
a) Yes, KRAV certified production
b) Yes, EU organic certified production
c) No.

E. **What breeds do you keep?**
a) Three breed cross with Hampshire
b) Three breed cross with Duroc
c) Purebred Landrace sows
d) Purebred Yorkshire sows
e) Other breedd or crosses (please describe)

Nursery Pigs

The following questions regard nursery pigs, from weaning until sold or moved to the finishing pig stable.

1. **Are there nursery pigs present on this site?**
a) Yes
b) No, go to question 53.

1a. **How many nursery pig places do you have?**

2. **What year did you start with nursery production?**

3. **On average, how old are the pigs when entering the nursery?**

4. **On average, how heavy are the pigs when entering the nursery?**

5. **On average, how old are the pigs when moving from the nursery?**

6. **On average, how heavy are the pigs when moving from the nursery?**

7. **Have you ever seen nursery pigs with tail damages, that you believe origins from tail biting, in the growing stables?**
   a) Yes
   b) No, move on to question 13.

8. **How often do you see nursery pigs that have been tail bitten (newly bitten)?**
   a) At least once/week
   b) At least once/month
   c) 3-6 times /year
   d) Twice a year or more seldom

8e. **In how many of the growing pens did you observe tail biting at the last tail-biting outbreak?**

9. **What do you think was the reason for the last tail biting outbreak in the growing stable?**

10. **Which do you think is the overall most common cause for tail biting in the growing stable?**

11. **If you discover tail biting in the growing stable, what do you usually do to prevent an outbreak? (More than one option can be chosen)**
   a) Remove the biter
   b) Remove the bitten pig
   c) Provide more straw
   d) Check the feeding system
   e) Check the ventilation
   f) Other (please the describe)

12. **How do you treat tail bitten pigs? (More than one option can be chosen)**
   a) Antibiotics, the pig is kept in the home pen.
   b) Antibiotics, the pig is moved to a sick pen.
   c) The pig is moved to a sick pen, but antibiotics are only provided if the pig has an impaired health status.
   d) No treatment
   e) Other treatment (please describe)

13. **If it had been legal to dock tails, according to the exception in the EU legislation, would you want to tail dock?**
   a) Yes

b) No

Straw usage to nursery pigs

14. **Do you provide straw to your nursery pigs?**
a) Yes, go to question no 16.
b) No

15. **If no, what is used instead?**
a) Wood shavings
b) Saw dust
c) Pite
d) Other (please describe)

15e. **If no, is there a special reason for using this material instead?**

16. **Do you use any other material in addition to straw in the nursery pens?**
a. Yes
b. No, go to question 18.

17. **If yes, which other material is used as well?**
a) Wood shavings
b) Saw dust
c) Pite
d) Other (please describe)

17e. **If yes, why is other material used in the nursery pens?**

18. **Do you use other stuff to occupy the nursery pigs other than straw? (such as chains, wood or plastic balls)**
a) Yes, go to question 19.
b) No

18a. **If yes, please describe.**

19. **Which type(s) of straw have you used in nursery pens (more than one answer may be selected)?** ***Skip question if straw is not used.***
a) Wheat
b) Oat
c) Rye
d) Barley
e) Other (please specify)

20. **Which type of straw do you prefer to use in nursery pens? (Choose one)** ***Skip question if straw is not used.***
   a) Wheat
   b) Oat
   c) Rye
   d) Barley
   e) Other (please specify)
   f) No preference

20a. **Why is that type of straw preferred? (Please explain)** ***Skip question if straw is not used.***

21. **Is there a type of straw you dislike using in nursery pens? (more than one answer may be selected)** ***Skip question if straw is not used.***
   a) Wheat
   b) Oat
   c) Rye
   d) Barley
   e) Other (please specify)
   f) Do not dislike using any type

21a. **Why? (please explain)** ***Skip question if straw is not used.***

22. **In what form to you usually store your bulk straw?** ***Skip question if straw is not used.***
   a) Large round bales
   b) Large square bales
   c) Small square bales
   d) Loose straw
   e) Other (please specify)

22f. **If bales are used, give an estimate of how much a bale weighs, in kg.** ***Skip question if straw is not used.***

23. **Is the straw chopped before use in the nursery pens?** ***Skip question if straw is not used.***
   a) Yes
   b) No, go to question 26.

24. **If yes, how is the straw treated?** ***Skip question if straw is not used.***
   a. Chopped
   b. Teared
   c. Other (Please describe)

25. **If yes, what is the approximate length of the straw used in the nursery? (in centimeters) *Skip question if straw is not used.***

26. **How often is straw added to the pens in the nursery? *Skip question if straw is not used.***
    a) Once/day
    b) Twice/day
    c) Other (please describe)

27. **Give an estimate, in kilograms, of how much straw is added to one nursery pen in one day. *Skip question if straw is not used.***

28. **Give an estimate of how much straw is used each week in the entire nursery. (Can be estimated as either number of bales used or number of tons used each week) *Skip question if straw is not used.***

29. **How is the straw distributed in the nursery pens? *Skip question if straw is not used.***
    a) Spread evenly over the floor of the pen
    b) Dropped in clumps on the floor of the pen for the pigs to break apart
    c) Placed in racks or troughs in the pen
    d) Other (Please describe)

30. **Do you ever alter the amount of straw used in a nursery pen based on the health of the pigs in the pen? *Skip question if straw is not used.***
    a) Yes
    b) No, go to question 32.

31. ***Skip question if straw is not used.***
    If yes, please describe:

32. **Approximately how many man-hours are spent each day distributing straw to the nursery pens? *Skip question if straw is not used.***

33. **How often do you scrape the pens in the nursery unit?**
    a. Twice a day
    b. Once a day
    c. Every second day
    d. Once a week
    e. When needed

34. **If there had been no limitations would have wanted to provide more straw to pigs in the nursery? *Skip question if straw is not used.***
    a) Yes
    b) No, go to question 36.

35. **If yes, what limitation(s) is/are preventing you from providing more straw to the pigs in the nursery? (May choose more than one) *Skip question if straw is not used.***
a) I have limited access to straw
b) It would require too much labor
c) It would be too costly
d) More straw would block the slatted floor
e) More straw would cause problems for the manure handling system
f) Other (Please describe)

<u>Pig Housing and Feeding System</u>

For the following questions about housing nursery pigs, if more than one type/size of pen/flooring is found in the barn, please describe the most common type you have.

36. **What is the approximate size, in square meters, of the pens in the nursery?**

37. **On average, how many pigs are housed in each nursery pen?**

38. **Is any part of the flooring in the nursery pens slatted?**
a) Yes
b) No, go to question 45.

39. **If yes, approximately what how much of a nursery pen floor is slatted? (in %)**
a) 30% or more than 20%
b) Approximately 20%
c) Less than 20%
d) Other share

40. **If yes, what material are the slats made of?**
a) Concrete
b) Plastic
c) Metal/cast iron
d) Other (please specify)

41. **If yes, what is the approximate width, in millimeters, of the slat openings?**

42. **If yes, what is the approximate length, in centimeters, of the slat openings?**

43. **If yes, what is the approximate width, in millimeters, of the slats?**

44. **If yes, what is the approximate length, in centimeters, of the slats?**

45. **How are the nursery pigs fed?**
    a) Liquid feed in trough
    b) Dry feed in trough
    c) Dry feed in automat
    d) Dry feed on floor
    e) Other (Please describe)

46. **If feed troughs are used in the nursery, approximately how long are they, in meters?**

47. **How is the nursery pig feed delivered**?
    a) Automatic feeding
    b) Hand feeding
    c) Other (Please describe)

48. **How many times a day do you feed the pigs in the nursery?**
    a) Ad libitum
    b) Once per day
    c) Twice per day
    d) Three times per day
    e) Other (Please describe)

Manure Handling System

49. **What type of manure removal system is used in the nursery?**
    a) Rope/cable with arm scraper(s)
    b) Rope/cable with box scraper
    c) Hydraulic scraper
    d) Shallow pits with pull-plugs
    e) Deep straw bedding
    f) Other (Please describe)

50. **How often is manure removed from under the pens in the nursery rooms?**
    a) Twice each day
    b) Once each day
    c) Every other day
    d) Other (Please describe)

51. **How frequently does straw cause problems (stoppages, blockages, back-ups etc.) in your manure removal system in the nursery? *Skip question if straw is not used.***
   a) Never
   b) A few times each year
   c) At least once/month
   d) At least once/week
   e) Every day

52. **What is your most common manure handling problem caused by straw use in the nursery unit? *Skip question if straw is not used.***

Finishing Pigs

The following questions pertain to finishing pigs (pigs from approximately 12 weeks old until market weight).

Are there finishing pigs present on this site? (If no, end survey)
a) Yes
b) No

53. **When did you start with finishing pig production?**

54. **How many finishing pig spaces do you have?**

55. **How many pigs are delivered to slaughter each year?**

56. **On average, how old are the pigs when they enter the finishing barn?**

57. **On average, how heavy are the pigs when they enter the finishing barn?**

<u>Tail biting</u>

58. **Have you ever seen finishing pigs with tail damages, that you believe origins from tail biting, in the finishing stables?**
   a) Yes
   b) No, go to question 66.

59. **If yes, approximately how many percentages of finishing pigs are tail bitten each batch?**

60. **If yes, in how many of the finishing pig pens did you observe tail biting at the last tail-biting outbreak?**

61. **If yes, how often do you see finishing pigs that have been tail bitten (newly bitten)?**
a) At least once/week
b) At least once/month
c) 3-6 times /year
d) Twice a year or more seldom

62. **What do you think was the reason for the last tail-biting outbreak?**

63. **Which do you think is the most common cause for tail-biting outbreaks in the finishing pig stable?**

64. **If you discover tail biting in the finishing pig stable, what do you usually do to prevent an tail-biting outbreak? (More than one option can be chosen)**
a) Remove the biter
b) Remove the bitten pig
c) Provide more straw
d) Check the feeding system
e) Check the ventilation
f) Other (please describe)

65. **How do you treat tail bitten pigs? (More than one option can be chosen)**
a) Antibiotics, the bitten pig is kept in the home pen.
b) Antibiotics, the pig is moved to a sick pen.
c) The pig is moved to a sick pen, but antibiotics are only provided if the pig has an impaired health status.
d) No treatment
e) Other treatment

66. **If it had been legal to dock tails, according to the exception in the EU legislation, would you want to tail dock?**
a) Yes
b) No

<u>Straw usage</u>

67. **Do you provide straw to your finishing pigs?**
a) Yes, go to question 70.
b) No

68. **If no, what is used instead?**
a) Wood shavings
b) Saw dust
c) Pite
d) Other (please describe)

69. **If no, is there a special reason for using this material instead?**

70. **Do you use any other material in addition to straw in the finishing pig pens? *Skip question if straw is not used.***
a. Yes
b. No, go to question 73.

71. **If yes, which other material is used as well? *Skip question if straw is not used.***
a) Wood shavings
b) Saw dust
c) Pite
d) Other (please describe)

72. **If yes, why is other material used? *Skip question if straw is not used.***

73. **Do you use other stuff to occupy the finishing pigs than straw? (such as chains, wood or plastic balls) *Skip question if straw is not used.***

73a. **If yes, please describe. *Skip question if straw is not used.***

74. **Which type(s) of straw have you used in the finishing pig pens (more than one answer may be selected)? *Skip question if straw is not used.***
a) Wheat
b) Oat
c) Rye
d) Barley
e) Other (Please specify)

75. **Which type of straw do you prefer to use in finishing pig pens? (choose one) *Skip question if straw is not used.***
a) Wheat
b) Oat
c) Rye
d) Barley
e) Other (please specify)
f) No preference

75g. **Why? (Please explain)** ***Skip question if straw is not used.***

76. **Is there a type of straw you dislike using in finishing pens? (May choose more than 1 answer)** ***Skip question if straw is not used.***
    a) Wheat
    b) Oat
    c) Rye
    d) Barley
    e) Other (please specify)
    f) Do not dislike using any type

76g. **Why? (Please explain)** ***Skip question if straw is not used.***

77. **In what form to you usually store the bulk straw used in finishing pens?** ***Skip question if straw is not used.***
    a) Large round bales
    b) Large square bales
    c) Small square bales
    d) Loose straw
    e) Other (Please specify)

78. **If bales are used, give an estimate of how much a bale weighs, in kg.** ***Skip question if straw is not used.***

79. **Is the straw chopped before use in the finishing pig pens?** ***Skip question if straw is not used.***
    a) Yes
    b) No

80. **If yes, what is the approximate length, in centimetres, of the straw used in the finishing pig pens?** ***Skip question if straw is not used.***

81. **How often is straw added to the finishing pig pens?** ***Skip question if straw is not used.***
    a) Once/day
    b) Twice/day
    c) Other (please describe)

82. **Give an estimate of how much straw is added to one finishing pig pen in one day.** ***Skip question if straw is not used.***

83. **Give an estimate of how much straw is used each week in the entire finishing pig production. (Can be estimated as either number of bales used or number of tons used each week)** ***Skip question if straw is not used.***

84. **How is the straw distributed in the finishing pig pens? *Skip question if straw is not used.***

a) Spread evenly over the floor of the pen
b) Dropped in clumps on the floor of the pen for the pigs to break apart
c) Placed in racks or troughs in the pen
d) Other (Please describe)

85. **Do you ever alter the amount of straw used in a finishing pig pen based on the health of the pigs in the pen? *Skip question if straw is not used.***

a) Yes
b) No, go to question 86.

85a. **If yes, please describe. *Skip question if straw is not used.***

86. **Approximately how many man-hours are spent each day distributing straw to the finishing pig pens? *Skip question if straw is not used.***

87. **How often is manure/dirty bedding removed from the finishing pig pens?**

a) Twice a day
b) Once a day
c) Every second day
d) Other interval (please describe)

88. **If there were no limitations, would you like to provide more straw to the finishing pigs? *Skip question if straw is not used.***

a) Yes
b) No, go to question 90.

89. **If yes, what limitation(s) is/are preventing you from providing more straw to the finishing pigs? (May choose more than one)**

a) I have limited access to straw
b) It would require more labor
c) It would increase costs too much
d) More straw would block the slatted floor
e) More straw would cause problems for the manure handling system
f) Other (Please describe)

<u>Pig Housing and Feeding System</u>

For the following questions about the housing of finishing pigs, if more than one type/size of pen / flooring is found in the barn, please describe the most common type you have.

90. **What is the approximate size, in square meters, of the finishing pig pens?**

91. **On average, how many pigs are housed in each finishing pig pen?**

92. **Is any part of the flooring in the finishing pig pens slatted?**
a) Yes
b) No, go to question 99.

93. **If yes, approximately what percent of a finishing pig pen floor is slatted?**

94. **If yes, what material are the slats made of?**
a) concrete
b) plastic
c) metal/cast iron
d) other (please specify)

95. **If yes, what is the approximate width, in millimeters, of the slat openings?**

96. **If yes, what is the approximate length, in centimeters, of the slat openings?**

97. **If yes, what is the approximate width, in millimeters, of the slats?**

98. **If yes, what is the approximate length, in centimeters, of the slats?**

99. **How are the finishing pigs fed?**
a) liquid feed
b) dry feed in trough
c) dry feed in automat
d) dry feed on floor
e) other (Please describe)

100. **If feed troughs are used in the finishing pig unit, approximately how long are they, in meters?**

101. **How is the finishing pig feed delivered?**
a) automatic feeding
b) hand feeding
c) other (Please describe)

102. **How many times a day do you feed the finishing pigs?**

a) Ad libitum
b) once per day
c) twice per day
d) three times per day
e) other (Please describe)

__Manure Handling System__

103. **What type of manure removal system is used in the finishing pig stable?**

a) Rope/cable with arm scraper(s)
b) Rope/cable with box scraper
c) Hydraulic scraper
d) Shallow pits with pull-plugs
e) Deep straw bedding
f) Other (Please describe)

104. **How often is manure removed from under the pens in the finishing pig units?**

a) Twice a day
b) Once a day
c) Every second day
d) Other interval (Please describe)

105. **How frequently does straw cause problems (stoppages, blockages, back-ups etc.) with your manure removal system in the finishing pig units?** ***Skip question if straw is not used.***

a) Never
b) A few times each year
c) At least once/month
d) At least once/week
e) Every day

106. **What is your most common manure handling problem caused by straw use in the finishing pig units?** ***Skip question if straw is not used.***
